# Proximal splenic artery embolization using a vascular plug in grade IV or V splenic trauma – a single centre 11-year experience

**DOI:** 10.1186/s42155-022-00345-8

**Published:** 2023-01-11

**Authors:** Samuel S. Xu, Kevin Eng, Fabio Accorsi, Derek W. Cool, Daniele Wiseman, Amol Mujoomdar, Leandro Cardarelli-Leite

**Affiliations:** 1 University of Western Ontario, London, Ontario Canada; 2 Halton Healthcare Services, 3001 Hospital Gate, Oakville, ON L6M 0L8 Canada

## Introduction

Splenic injury is one of the most common injuries following blunt abdominal trauma, reported in 32% of trauma cases (Lynn et al., 2009). The non-operative management (NOM) of hemodynamically stable patients has become the standard of care and splenic artery embolization (SAE) is being used as an adjunct to observation to increase the success rate of NOM (Habash et al., 2021; Patil et al., 2020).

SAE is a well documented procedure for splenic trauma, originally described by Sclafani in 1981 (Sclafani, 1981), offering a splenic preservation treatment (Habash et al., 2021; Patil et al., 2020; Haan et al., 2005; Brahmbhatt et al., n.d.; Olthof et al., 2017; Ahuja et al., 2015; Cretcher et al., 2021). The technique of proximal embolization, distal embolization, or combined embolization is well described in the literature, with comparable clinical outcomes (Jambon et al., 2018; Frandon et al., 2014; Wong et al., 2017; Gheju et al., 2013; Quencer & Smith, 2019; Cinquantini et al., 2018) and overall good splenic preservation. Generally, proximal embolization is performed for global splenic trauma to decrease the perfusion pressure to the spleen, while distal embolization is used to embolize more focal injuries (Jambon et al., 2018; Quencer & Smith, 2019; Johnson et al., 2021). However, heterogeneity of technique varies in both the literature and in practice (Habash et al., 2021; Haan et al., 2005; Ahuja et al., 2015; Quencer & Smith, 2019; Demetriades et al., 2012; Banerjee et al., 2013).

The main purpose of this study was to retrospectively assess if proximal SAE with a vascular plug alone is effective in stopping bleeding and achieving splenic salvage for the treatment of grade IV or V splenic injuries. Furthermore, complication and mortality rates were compared between the use of additional coil embolization versus vascular plug alone.

## Materials and methods

A retrospective review was performed to include all splenic artery embolization procedures performed at the local tertiary care level I trauma centre, between November 2010 to January 2021. Institutional Research Ethics Board approval was obtained. Informed consent was waived by the review board for this study.

**Fig. 1:**
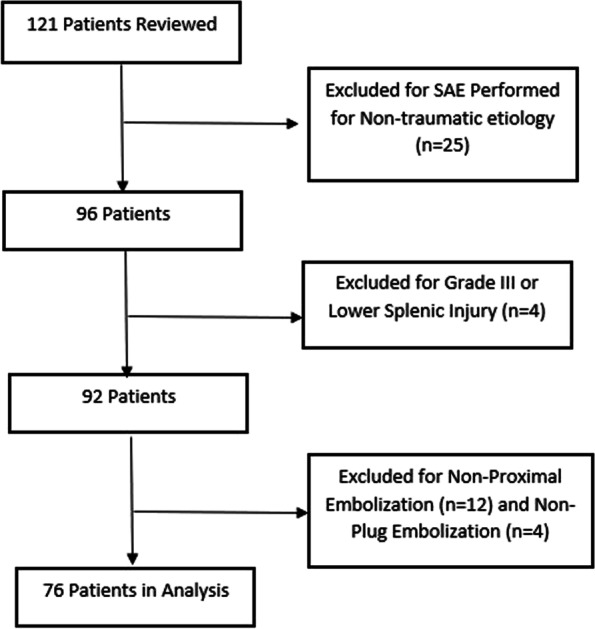
Patient inclusion

Exclusion criteria included lack of pre-procedural imaging, splenic embolization procedure performed for an indication other than blunt abdominal trauma, distal embolization, non-vascular plug embolization, and splenic trauma grade III or lower (Fig. 1). The most widely accepted grading classification of the splenic injuries is the American Association of the Surgery of Trauma (AAST) splenic injury scale (Kozar et al., 2018) (Table 1) was used for injury grading. Patients included in this study were hemodynamically stable, as hemodynamically unstable patients were routinely taken to the operating room as per the local standard practice. The CT for all patients were reviewed retrospectively by abdominal and interventional radiologists not involved in the procedures, blinded to the clinical outcomes, and splenic injury was graded using the AAST splenic injury scale (Kozar et al., 2018).

**Table 1:** Summary of 2018 AAST spleen injury scale for CT Findings

AAST Grade	CT Imaging Criteria
**I**	Subcapsular hematoma < 10% surface area; parenchymal laceration < 1 cm depth or capsular tear
**II**	Subcapsular hematoma 10–50% surface area or hematoma < 5 cm; parenchymal laceration 1–3 cm
**III**	Subcapsular hematoma > 50% surface area or ruptured subcapsular or intraparenchymal hematoma ≥5 cm; parenchymal laceration > 3 cm depth
**IV**	Presence of splenic vascular injury or active bleeding confined to the spleen; laceration with vessel involvement resulting in > 25% devascularization
**V**	Presence of splenic vascular injury with active bleeding beyond the spleen; shattered spleen

The time between the acquisition of the initial CT (considered time of diagnosis of the splenic injury) to the time to the start of the embolization procedure was calculated in hours. Splenic artery diameter and the presence of splenic pseudoaneurysms and its size were recorded. Procedural details including the type and diameter of the plug and coils (if used) were recorded. Radiation dose, if available due to change in software and infrastructure resulting in loss of data, was also recorded.

Technical success was defined as completion of the procedure with successful proximal deployment of the plug with decreased flow through the splenic artery. Clinical success was defined as the cessation of bleeding and maintained hemodynamic stability within 30 days of the embolization. All adverse events were documented and defined by the Society of Interventional Radiology Adverse Event Classification (Kozar et al., 2018). New left sided pleural effusions were looked at specifically as an indicator for splenic inflammation and irritation, which is a described association in the literature of SAE for hypersplenism (Araten et al., 2014). Any follow-up imaging, as well as follow up clinical notes, were reviewed.

### Demographics and Preprocedural data

A total of 121 patients underwent splenic embolization. Patients with non-traumatic etiology (*n* = 25), non-proximal embolization (*n* = 12), grade III splenic injury or lower (*n* = 4), and non-plug embolization (n = 4) were excluded, leaving 76 study patients. Preprocedural details are summarized in Table 2. On preprocedural CT, pseudoaneurysms were present in 64 of the cases (84.2%). The most frequently reported number of pseudoaneurysms was more than five, totaling 26 cases (34.2%).

**Table 2:** Preprocedural details including CT findings

	Number of Patients or Mean Values
**Mean Age (years)**	42.3 ± 17.0
**AAST Grade**	
*Grade 4*	73 (96.1%)
*Grade 5*	3 (3.1%)
**Mean Splenic Artery Diameter (mm)**	6.4 ± 1.1
**Pseudoaneurysm Present**	64 (84.2%)
**Mean Size of Pseudoaneurysms (mm)**	9.2 ± 4.7
**Number of Pseudoaneurysms**	
*One*	8 (10.5%)
*Two*	10 (13.2%)
*Three*	3 (3.9%)
*Four*	17 (22.4%)
*More than Five*	26 (34.2%)
**TRAUMA EVENT**	
*Motor vehicle collision*	37
*Fall*	20
*motorcross/atv/snowmobile*	9
*Assault*	5
*other*	5

### Technique

#### CT technique

**Fig. 2:**
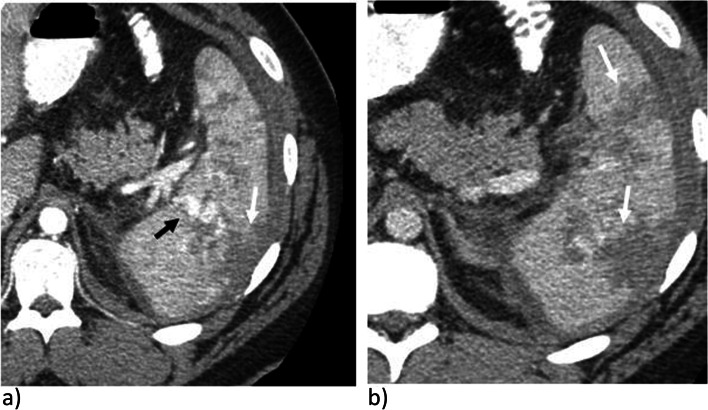
CT in a trauma patient in a) mixed arterial and portal venous phase in a dual bolus injection and b) delayed phases demonstrating a grade IV splenic injury with large lacerations (solid white arrow) and pseudoaneurysms (solid black arrow). There is no extravasation of contrast as confirmed on

CT were acquired on 64 slice CT scanners (GE Lightspeed, Boston, MA, USA) between November 2010 to October 2019. Subsequently, all images were acquired on 128 or 320 slice CT scanners (Canon Aquilion, Tokyo, Japan). CT protocol during initial trauma acquisition of the thorax, abdomen, and pelvis was performed following injection of a dual bolus IV contrast technique. Initial injection of 90 cc of Omnipaque 350 is given, followed by 15 cc of normal saline, both at 3 cc/sec. Following a 15 second pause, a 50 cc injection of Omnipaque 350 is performed with a 20 cc injection of normal saline, both at 4 cc/sec (Fig. 2a). A check on the scanner is then performed by the radiologist or radiology resident on call for decision of obtaining delayed images, acquired at 5 minutes post injection (Fig. 2b). CT parameters are set at kV 120, helical pitch 65, rotation time 0.35 seconds, 0.5 mm slice thickness with 2 mm thick reformats. Follow up imaging includes CT of the abdomen and pelvis with similar CT parameters. Non-contrast, arterial (25 seconds) and portal venous (55 seconds) phase acquisitions are acquired following injection of 100 cc of Omnipaque 350 with 20 cc of saline at 4 cc/sec.

#### Procedural technique

**Fig. 3:**
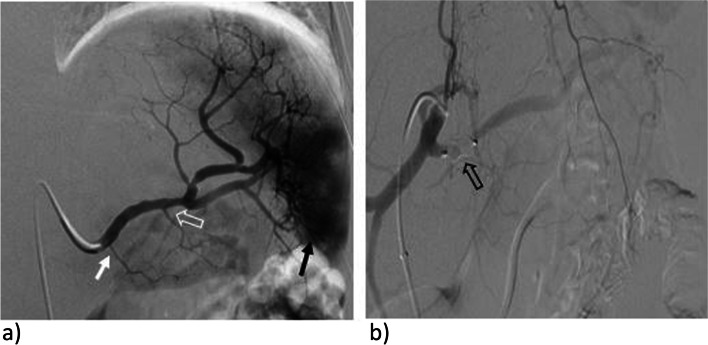
Embolization technique. **A** Digital subtraction angiography (DSA) of the splenic artery after selective catheterization with a C2 catheter acquired in the same patient prior to embolization. Region of hyperemia in the lower spleen (solid black arrow) and suspected pseudoaneurysms corresponding to the region of grade IV injury. Note the origin of the dorsal pancreatic artery (solid white arrow) and the greater pancreatic artery (open white arrow). **B** DSA of the celiac axis following proximal embolization. A vascular plug (open black arrow) has been deployed, covering the origin of the dorsal pancreatic artery and slightly proximal to the origin. There is decreased flow in the splenic artery, without residual hyperemia or pseudoaneurysms

Procedures were performed by six different interventional radiologists. Access was through the common femoral artery under ultrasound guidance. A combination of 7 or 8-Fr valved RDC guiding catheters (Vista Brite; Cordis, FL, USA) or 6 Fr Raabe sheaths (Cook Medical, IND, USA) and 5-Fr Cobra 2 or Sim 1 catheters were used to select the splenic artery (Fig. 3a). The vascular plugs were oversized by 50–100% in relation to the diameter of the splenic artery measured on pre-procedural imaging and then deployed proximally within the splenic artery, covering the origin of the dorsal pancreatic artery (Fig. 3b). This location was chosen since plugs tend to be challenging to navigate distally through tortuosity.

The Amplatzer’s type I (AVP; Abbot, IL, USA) plugs ranging from 8 to 12 mm in size were used in most cases. In two occasions, a Micro Vascular Plug (MVP; Medtronic, Ireland) was deployed through a 2.8-Fr microcatheter (Progreat; Terumo, Japan) due to difficulty in placing a sheath into the splenic artery. Plug and coil sizes were determined based on pre-procedural CT measurements. In most cases, the operators did not wait for complete angiographic occlusion of the splenic artery following plug deployment to terminate the procedure. However, some operators decided to add a coil proximal to the plug to achieve faster occlusion. Closure of the femoral puncture was with either a closure device or manual compression, which was case and operator dependent.

#### Statistical analysis

The data, including adverse event rates, radiation dose, mortality, and periprocedural data, was analyzed using the appropriate statistical tests on SPSS Software (Version 25, IBM, Armonk NY, USA). Analysis between categorical groups was performed using a Chi-square test. If nominal data was analyzed with comparison of means, *t* tests were utilized. *p* < 0.05 was considered statistically significant.

## Results

### Procedural data and outcomes

Procedural and post procedural details are summarized in Table 3. The technical success rate was 100%. Clinical success rate was achieved in 72/76 patients (94.7%). Splenic preservation was successful in 73/76 patients (96.1%). Within the total study population, moderate to severe adverse events occurred in 12 patients (15.8%), of which 7/12 were new splenic infarctions < 33% of the parenchyma (9.2%). The other adverse events included puncture site pseudoaneurysm (1/12), splenic hematoma (1/12), and persistent splenic pseudoaneurysms or bleeding resulting in patient hemodynamic instability necessitating splenectomy (3/12). New left sided pleural effusions developed in 8/76 of the patients (10.5%) following the procedure, that resolved prior to discharge without intervention. Overall mortality of the patients was 2.6% (2/76 patients), with one patient succumbing to other injuries not related to their splenic trauma. However, the cause of death in the patients was secondary to the polytrauma, and not directly related to the splenic embolization procedure.

**Table 3:** Procedural and post procedural details

procedural and follow-up details	Number of Patients or Mean Values
**Time to Procedure from CT (hours)**	10.9 ± 14.6
**Additional coil embolization**	18 (23.7%)
**average coil size (mm)**	10.7 ± 1.5
**average plug size (mm)**	10.8 ± 1.4
**moderate to severe adverse events**	
*Splenic Infarct*	14 (14.6%)
*Other*	3 (3.1%)
**New Left Pleural Effusion**	11 (11.5%)
**Mortality**	4 (4.2%)
**follow-up ct available**	35
*time to follow-up CT (DAys)*	422 ± 860
*Minimum time to follow-up CT (days)*	0
*maximum time to follow-up CT (days)*	3259

### Comparison of vascular plug alone and additional coil embolization

There was a statistically significant difference between the rate of adverse events between the two embolization groups. Moderate to severe adverse events occurred in 6/58 patients (10.3%) with vascular plug alone, and 6/18 patients (33.3%) with combination embolization (*p* = 0.03), as presented in Table 4. There was no statistically significant difference between the two groups for mortality rate (1/58 vs. 1/17; *p* = 0.42) or the development of a new left sided pleural effusion (6/58 vs. 2/18; *p* = 0.61). There was no statistically significant difference for the time to embolization, if pseudoaneurysms were present on the preprocedural CT, number of pseudoaneurysms present, or grade of the splenic trauma (all *p* > 0.05). The radiation dose was available in 31 cases, which showed no difference between the two groups, with mean dose with the use of plug alone 0.5 ± 0.5 Gy and combination embolization 0.5 ± 0.4 Gy (*p* = 0.17).

**Table 4:** Complications between vascular plug and vascular plug with coil embolization

moderate to severe adverse events	Vascular Plug (*n* = 58)	Vascular Plug + Coil (*n* = 18)
*Splenic Infarct*	**4**	**3**
*Puncture site pseudoaneurysm*	**0**	**1**
*Hematoma*	**1**	**0**
*Persistent bleeding*	**0**	**2**
*Persistent pseudoanuerysm*	**1**	**0**

## Discussion

This study offers a new perspective in proximal splenic artery embolization, with the use of plug alone in comparison to combination embolization with additional coils. There is a significant decrease in adverse events with the use of vascular plug alone, that contributes to overall splenic preservation. The importance of the spleen in maintaining the body’s immune system and antibody production is well known, with knowledge that open splenectomy is strongly associated with systemic infection (Demetriades et al., 2012). Therefore, splenic preservation treatment options are important in the management of splenic trauma, with splenic artery embolization widely accepted as a safe and effective treatment in patients with hemodynamically stable grade III or higher splenic injury (Haan et al., 2005; Brahmbhatt et al., n.d.; Cretcher et al., 2021; Quencer & Smith, 2019). Technical success was achieved in all patients in this study with grade IV or higher splenic trauma, comparable to the rates described in the literature (Habash et al., 2021; Frandon et al., 2014; Cinquantini et al., 2018). Cessation of bleeding and maintained hemodynamic stability occurred in 94.7% of the patients, comparable to literature rates of both distal and proximal SAE (Habash et al., 2021; Frandon et al., 2014; Banerjee et al., 2013; Wu et al., 2008; Ekeh et al., 2013). The splenic salvage rate in this study was 96.1%, comparable to the reported literature rates in proximal SAE with the use of coil and plug embolization (Habash et al., 2021; Jambon et al., 2018; Quencer & Smith, 2019; Zhu et al., 2011).

Combined embolization, such as with plug and coil in proximal SAE, is described in the literature (Habash et al., 2021; Patil et al., 2020; Brahmbhatt et al., n.d.; Quencer & Smith, 2019; Zhu et al., 2011). However, there are no dedicated analyzes comparing the use of combined embolic agents to single embolic agents. Due to local practice, it was possible to compare the outcomes in proximal splenic artery embolization with the use of a plug versus plug and coils. Interestingly, this demonstrated a statistically significant increased adverse event rate in the group of patients that had embolization with both plug and coils (*p* = 0.036). The most common adverse event was splenic infarct. One postulation for the increased rate of splenic infarct may be due to the theoretical faster time to complete occlusion with the addition of coils. As a result, collateral vessels may not be established to perfuse the spleen in certain patient populations, resulting in ischemia, and ultimately infarct, of the spleen. Additionally, with addition of coils, the landing zone of the embolic agents is longer, which could result in the occlusion of the ostia of potential collateral vessels to the spleen. Other factors could be involved, but further studies would be required to establish this relationship. A potential benefit of proximal SAE with plugs alone could include a “plug and forget” approach where the operator would deploy the plug without having to chase it with a coil or wait for complete stasis of flow to terminate the intervention. This offers reassurance in the procedural outcomes, such that there should theoretically be a decrease in need for additional radiation with need for less repeat angiographic images and fluoroscopy time following deployment of the plug. Furthermore, the procedural time should also theoretically decrease in this approach as the need for further embolization was shown to not needed to achieve a desired outcome of hemodynamic stability in this study.

The literature reported adverse event rates up to 20–29% for major adverse events, which include splenic infarct, splenic abscess, and continued bleed necessitating open splenectomy (Habash et al., 2021; Wu et al., 2008; Ekeh et al., 2013). This is comparable to moderate to severe adverse events per the new SIR guidelines, and the rate of moderate to severe adverse events is lower with proximal SAE than distal SAE as described in several reviews (Brahmbhatt et al., n.d.; Frandon et al., 2014; Wong et al., 2017; Quencer & Smith, 2019; Cinquantini et al., 2018). The most common adverse event in this study was splenic infarct, occurring in seven of the patients (9.2%). Three patients required open splenectomy within 1–5 days following embolization, which was documented as severe adverse events, but are considered treatment failure rather than a complication of the procedure.

Following SAE, the development of a left-sided pleural effusion has been described, thought to be from irritation of the diaphragm from inflammation in upper pole splenic embolizations (Habash et al., 2021; Cinquantini et al., 2018; Araten et al., 2014; Wu et al., 2008; Ekeh et al., 2013). This is a known adverse event described in SAE for hypersplenism (Araten et al., 2014), and this was investigated in this study as a surrogate marker for degree of splenic inflammation that SAE caused. This occurred in eight of the patients in this study (10.5%), which is lower than Wu et al (Wu et al., 2008) (33%) that had distal embolization patients and Ekeh et al (Ekeh et al., 2013) (17%) that saw a similar number in both proximal, distal, and combined SAE. There was no statistically significant difference in the rate of left pleural effusion based on embolization techniques in this study. The overall percentage of this adverse event was similar in literature reports, with a higher rate reported in distal upper splenic embolizations (Araten et al., 2014; Wu et al., 2008; Ekeh et al., 2013). Interestingly, the adverse event rates in this study were similar to the literature despite the proximal embolization location being placed over the dorsal pancreatic artery origin. This suggests good alternative collaterals are available proximal to this vessel origin to maintain flow to the surrounding solid organs. The mortality rate between the two groups in this study was not significantly different and were due to polytrauma with other injuries that the patient ultimately succumbed to.

In the setting of proximal SAE, the procedure time and radiation dose has been described in literature to be decreased in comparison to distal SAE (Quencer & Smith, 2019; Johnson et al., 2021; Zhu et al., 2011). In comparison of plug to coil, Zhu et al. (Zhu et al., 2011) found that the procedure time was not significantly different, with a trend toward decrease in the plug alone group. However, the radiation dose was significantly decreased with the use of a plug compared to coil for proximal SAE (Zhu et al., 2011). Similar results have been reported with other reviews and studies (Jambon et al., 2018; Johnson et al., 2021). Due to a migration of the data storage system at the local institute during the study data review period, the details of procedure length were not available for review, and the total radiation dose was only available in 31 cases. This showed no statistically significant difference between the use of plug and combination embolization, which is likely due to the small sample size.

There are limitations to this study. First, as a retrospective cohort review, the nature of the study leads to possible cofounding factors that are not fully accounted for in the analysis such as patient past medical history, medication history or associated traumatic injuries. Additionally, this is a single centre review, which could limit the range of external validity. Furthermore, not all data points where available for the entirety of the study population. Moreover, as the use of the embolic agents is up to the interventionist at the time of the procedure and not standardized, there may be other procedural factors not documented that could ultimately skew the results if the sicker or more complex patients were heavily represented in one group over the other.

Follow-up imaging was limited in this study, with only 35 patients getting CT scans in the same institution after the embolization. This would limit the assessment of the cessation of bleeding or resolution of the pseudoaneurysm from an imaging perspective. If the patient was clinically stable, the local clinical practice, and to appropriately utilize resources, favor to not further image the patient. This would not significantly limit the assessment of clinical success in this study, as patients that did not require further imaging were clinically stable, although there remains a possibility of under-representing persistent small pseudoaneurysms.

## Conclusion

Overall, this study supports the use of proximal SAE in high grade hemodynamically stable splenic injury with a single vascular plug for stopping bleeding and promoting splenic salvage. This study suggests there are increased adverse events in the use of combined embolization in proximal SAE compared to just plug embolization. However, larger studies and randomized trials would be helpful in further evaluating this association.

## Data Availability

The datasets generated and/or analysed during the current study are not publicly available due to local ethics restrictions, but certain anonymized data may be available from the corresponding author on reasonable request.

## Abbreviations

AAST: American Association of the Surgery of Trauma
NOM: Non-operative management
SAE: Splenic artery embolization
